# Designable Layer Edge States in Quasi‐2D Perovskites Induced by Femtosecond Pulse Laser

**DOI:** 10.1002/advs.202201046

**Published:** 2022-05-12

**Authors:** Yu Miao, Zeqi Xiao, Zeyu Zheng, Da Lyu, Qin Liu, Jieyu Wu, Yongbo Wu, Xiewen Wen, Lingling Shui, Xiaowen Hu, Kai Wang, Zhilie Tang, Xiao‐Fang Jiang

**Affiliations:** ^1^ Laboratory of Quantum Engineering and Quantum Material School of Physics and Telecommunication Engineering South China Normal University Guangzhou 510006 P. R. China; ^2^ Guangdong Provincial Key Laboratory of Nanophotonic Functional Materials and Devices School of Information and Optoelectronic Science and Engineering South China Normal University Guangzhou 510006 P. R. China; ^3^ Guangdong Provincial Key Laboratory of Optical Information Materials and Technology & Institute of Electronic Paper Displays South China Academy of Advanced Optoelectronics South China Normal University Guangzhou 510006 P. R. China; ^4^ Department of Mechanical Engineering City University of Hong Kong Tat Chee Avenue Kowloon Hong Kong; ^5^ Materials Research Institute Pennsylvania State University University Park PA 16802 USA

**Keywords:** femtosecond pulsed laser, laser ablation, layer edge states, photoluminescence, quasi‐2D perovskites

## Abstract

The low‐energy layer edge states (LESs) from quasi 2D hybrid perovskite single crystals have shown great potential because of their nontrivial photoelectrical properties. However, the underlying formation mechanism of the LESs still remains controversial. Also, the presence or creation of the LESs is of high randomness due to the lack of proper techniques to manually generate these LESs. Herein, using a single crystals platform of quasi‐2D (BA)_2_(MA)_n−1_Pb_n_I_3n+1_ (*n* > 1) perovskites, the femtosecond laser ablation approach to design and write the LESs with a high spatial resolution is reported. Fundamentally, these LESs are of smaller bandgap 3D MAPbI_3_ nanocrystals which are formed by the laser‐induced BA escaping from the lattice and thus the lattice shrinkage from quasi‐2D to 3D structures. Furthermore, by covering the crystal with tape, an additional high‐energy emission state corresponding to the reformation of (BA)_2_PbI_4_ (*n* = 1) within the irradiation region is generated. This work presents a simple and efficient protocol to manually write LESs on single crystals and thus lays the foundation for utilizing these LESs to further enhance the performance of future photoelectronic devices.

## Graphical abstract

Femtosecond laser ablation is proposed to write the layer edge states (LESs) with high spatial resolution and preciseness in quasi 2D perovskites. These low‐energy LESs are MAPbI_3_ nanocrystals formed by laser induced BA escaping from the lattice and the lattice shrinkage. Sealing of the irradiated film can further induce additional high‐energy states by the formation of (BA)_2_PbI_4_.

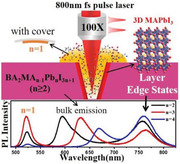

## 1 Introduction

Quasi‐two‐dimensional (quasi‐2D) perovskites, particularly the Ruddlesden−Popper halide perovskites,^[^ ^1^ ^]^ have shown great potential for the applications in photoelectronic such as light‐emitting diodes (LEDs),^[^ ^2^ ^]^ solar cells,^[^ ^3^ ^]^ and photodetectors^[^ ^4^ ^]^ due to the flexibility in modulating the exciton binding energy and bandgap, high quantum efficiency, and excellent moisture stability.^[^ ^1^, ^5^ ^]^ Recently, the low‐energy layer edge states (LESs) have been discovered in these quasi‐2D halide perovskites and display attractive photoelectric properties such as long carrier lifetime^[^ ^6^ ^]^ and high electrical conductivity.^[^ ^7^ ^]^ LESs provide a direct pathway for charge dissociation and thus the potential to significantly improve the performance of photoelectronic devices based on quasi‐2D perovskites.^[^ ^8^ ^]^

In the year of 2017, Blancon et al. firstly reported the LESs that exhibit a lower‐energy photoluminescence (PL) locating at the edge of exfoliated quasi‐2D perovskites (BA)_2_(MA)_n‐1_Pb_n_I_3n+1_ when the quantum well is thick, i.e., *n* ≥  3.^[^ ^6^ ^]^ They found that LESs can trap the photogenerated excitons and dissociate them into free carriers with longer lifetime and lower energy. In 2018 Feng et al. revealed that these LESs can efficiently boost the photocurrent and enhance the photo‐response in photodetectors using quasi‐2D perovskite single‐crystals.^[^ ^9^ ^]^ Wang et al. further demonstrated an extraordinary conductive feature along the LESs at room temperature and firstly visualized these states using conductive atomic force microscopy (c‐AFM) in 2019.^[^ ^7^ ^]^ These unexpected properties promote the LESs to be a crucial factor to further improve the device performance of quasi‐2D perovskites optoelectronics. In order to understand the formation mechanism of LESs, the intrinsic origin and external influence factors of LESs have been more deeply explored. In 2018, Kepenekian et al. proposed an elastic model, which claimed that the lattice mismatch may induce the formation of LESs.^[^ ^10^ ^]^ Zhang et al. provided a more profound understanding that the asymmetry in the chemistries of the iodine and Pb atoms at the edge may lead to the LESs.^[^ ^11^ ^]^ Recently in 2021, Hong et al. demonstrated that the LESs are stabilized by the ferroelectric alignment of organic cations.^[^ ^12^ ^]^ In addition to these internal factors, Shi et al. revealed that external factors such as moisture level can have an impact on the LESs formation.^[^ ^13^ ^]^ Zhao et al. reported that the LESs can be modulated by the MAI/BAI solution rinsing process,^[^ ^14^ ^]^ indicating the LESs are associated with the loss of BA binding ligands. Very recently, Qin et al. observed three‐dimensional (3D) MAPbBr_3_ spontaneously formed in the fresh edges of 2D (BA)_2_(MA)_2_Pb_3_Br_10_ created by physical cutting and milling.^[^ ^15^ ^]^ So far, it is still inconclusive whether intrinsic or extrinsic factor dominates the formation mechanism of LESs in quasi‐2D perovskites. Moreover, the generation of LESs is still a random phenomenon occurring in the process of material synthesis or the mechanical exfoliation of the single crystal. Therefore, there lacks a precise control of the LESs formation, let alone the manufacturing or programing of predesigned patterns of LESs.

To address these issues, by using a material platform of quasi‐2D (BA)_2_(MA)_n−1_Pb_n_I_3n+1_ (*n* = 1−4) single crystal flakes and a technique of femtosecond (fs) pulse laser direct writing, for the first time, we find that the LESs can be manually created by the laser ablation process on arbitrary regions of the crystal (*n* ≥ 2). Through a series of characterization techniques including time‐resolved photoluminescence (TRPL) spectroscopic mapping, scanning electron microscopy (SEM), and energy dispersive X‐ray spectroscopy (EDS) on various samples, we find that the laser‐generated LESs come from the 3D MAPbI_3_ perovskite nanocrystals formed at the edges of the irradiation trace after the loss or the replacement of BA ligands. In parallel, we demonstrate that the LESs can be precisely written by a microscopic fs laser ablation on quasi‐2D perovskites, which provides the foundation for the design and manipulation of the excited states in these materials.

## 2 Results and Discussion

**Figure 1:**
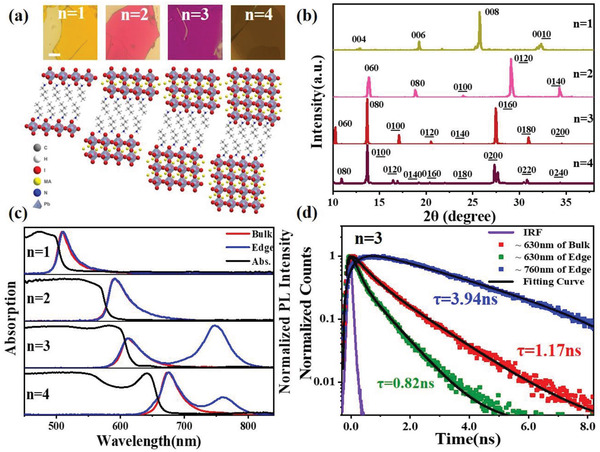
a) Optical images and the crystal structures of quasi‐2D single crystals of (BA)_2_(MA)_n‐1_Pb_n_I_3n+1_ (*n* = 1–4). b) The corresponding single crystal XRD patterns. c) UV–vis–NIR absorption and PL spectra from the bulk (red line) and edge (blue line) regions, respectively. d) Normalized PL decay dynamics of the bulk (≈630 nm) and the edge (≈630 and ≈760 nm) regions from the (BA)_2_(MA)_2_Pb_2_I_10_ (*n* = 3) perovskite single crystal.

The single crystals of quasi‐2D perovskites of (BA)_2_(MA)_n−1_Pb_n_I_3n+1_ (*n* = 1–4) are synthesized according to the solution–precipitation–reaction method.^[^ ^16^ ^]^ The details are described in the Experimental Section. For the microscopic measurements, we use the mechanical exfoliation method to obtain the 2D perovskite flakes (*n* = 1–4) with lateral dimension of >20 µm and thickness from tens of nanometers to a few micrometers, followed by transfer to a cover glass substrate. In order to isolate the sample from air, we use tape to encapsulate the sample. **Figure** **1**a shows the optical images and corresponding crystal structures of the (BA)_2_(MA)_n‐1_Pb_n_I_3n+1_ (*n* = 1–4) single crystals. The index n can be understood by the quantum well thickness of the material, while the organic barrier has a fix thickness corresponding to the thickness of the BA Van der Waals bilayer. Figure 1b displays the X‐ray diffraction (XRD) patterns of these samples, which show the peak positions of corresponding scattering plane distances consistent to the corresponding lattice parameters of the materials. Particularly, the single crystal flakes are directly transferred onto the flat substrates to maintain the original orientations when we carry out the XRD measurement. Compared to the simulated powder XRD patterns (Figure S1, Supporting Information), there are high intensity diffraction peaks along (00*l*) direction for *n* = 1 sample and (0*l*0) direction for *n* = 2–4 samples, as well as the absence of other peaks (e.g., (111) and (202)). These results are consistent to the sample alignment to the substrate (the quasi‐2D planes are in parallel to the substrate),^[^ ^16a^ ^]^ and the absence of other planes also agrees well with the single‐crystalline feature of the sample, suggesting a high phase purity.

Figure 1c shows the UV–vis–NIR absorption spectra of these mechanically exfoliated 2D perovskite flakes with *n* = 1−4. The energy bandgap of quasi‐2D perovskites decreases with the increase of the index number (*n*), i.e., the layer number of the 2D [PbI_6_] metal halide layer. This bandgap decrease can be ascribed to the decrease of the quantum confinement effect.^[^ ^5^, ^17^ ^]^ The PL spectra from the edge and bulk regions in Figure 1c confirms that the low‐energy LESs exist in single crystals of *n*  ≥  3 but are absent in *n* = 1 and 2, in agreement with the previous reports.^[^ ^6^, ^7^ ^]^ We find the PL spectra of LESs between *n* = 3 and 4 are almost same and also similar to the 3D MAPbI_3_ perovskites (*n* = ∞). Moreover, as shown in Figure 1d, for the *n* = 3 sample, the average PL lifetime at 630 nm in the bulk region is around 1.17 ns. The PL lifetime at the same wavelength (630 nm) in the edge region is around 0.82 ns, which is smaller than the bulk region. The low‐energy emission (760 nm) from edge states has a much longer lifetime (3.94 ns) and exhibits a relatively slow rising component (≈0.42 ns), indicating energy transfer and carrier filling from the higher‐energy intrinsic exciton to the low‐energy edge states.^[^ ^6^, ^14^ ^]^ Similar results are also observed in the *n* = 4 sample (Figure S2, Supporting Information). The fluorescence lifetime imaging microscopy (FLIM) results further verify the general existence of LESs in the crystal edges in these quasi‐2D (BA)_2_(MA)_n−1_Pb_n_I_3n+1_ perovskites when *n* ≥ 3 (Figure S3, Supporting Information).

**Figure 2:**
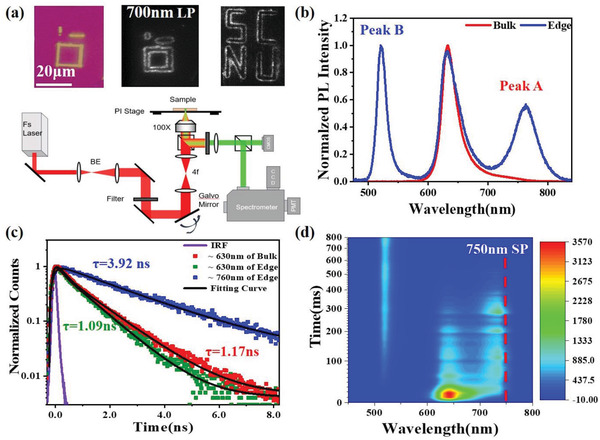
a) Optical and PL images collected at emission channels of >700 nm of (BA)_2_(MA)_2_Pb_3_I_10_ (*n* = 3) flakes after irradiated with 800 nm fs laser pulses (above) and the corresponding experimental setup scheme (below). b) PL spectra of the 2D perovskite crystal from the bulk and laser‐induced edge regions upon 400 nm excitation. c) Normalized PL decay dynamics of the bulk (≈630 nm) and the laser‐induced edge (≈630 and ≈760 nm) region. d) Time evolution of in situ two‐photon excited photoluminescence (TPPL) spectra upon fs pulse laser ablation.

To manually write a pattern of LESs, we employ a high‐power fs pulse laser with a wavelength of 800 nm for the ablation process. Surprisingly, these manually created LESs also exhibit the low‐energy emission at the laser‐induced edge regions in the single crystal samples of quasi‐2D perovskites (*n* ≥ 2). The fs laser pulses possess a pulse width of 200 fs and a repetition rate of 80 MHz. Figure S4 (Supporting Information) shows the correlation between the intensity of low‐energy emission emitted from the laser‐induced edge and the fs laser fluence upon an identical irradiation time of 50 ms. The low energy PL peak can only be observed when the laser fluence is beyond 11.7 mJ cm^−2^. The optical images indicate there is a structural damage of the bulk region of quasi‐2D perovskite. **Figure** **2**a shows the optical image and low‐energy PL (>700 nm) images of the *n* = 3 perovskite after patterning by fs laser ablation. The PL images are collected upon 395 nm wide‐field excitation and the emission signal is selected by a 700 nm long‐pass (LP) filter before the camera. The corresponding experimental scheme for fs laser ablation and PL imaging is also shown in Figure 2a. We find that the low‐energy emission can be observed at the edges of the laser‐irradiated trace. Figure 2b compares the PL spectra collected at both the bulk and laser‐induced edge region upon an excitation wavelength of 400 nm. The original bulk region only has an emission with a peak at ≈630 nm, while the laser‐induced edges exhibit an additional low‐energy emission (peak A) centered at ≈760 nm and a high‐energy emission (peak B) centered at ≈518 nm, accompanied with the original emission at ≈630 nm. The PL spectra of the lower‐energy peak A created by fs laser ablation is similar to the natural LESs emission. In order to understand the origin of these peaks, we also quantify the PL lifetime of the LESs acquired by fs laser ablation (Figure 2c). The PL lifetime of the lower‐energy peak A is 3.92 ns, which is identical to that of natural LESs created by mechanical exfoliation. In a particular comparison with the natural low‐energy LESs, the rising component of peak A is faster here, which indicates a more efficient energy transfer from the intrinsic exciton to the laser‐induced LESs. The PL lifetime distribution of laser‐induced LESs was also confirmed by the FLIM result, which shows the LESs can only be observed at the edge region of the laser ablation (Figure S5, Supporting Information). We speculate these fs laser‐induced LESs may be possibly introduced by the stoichiometric loss of BA component during the ablation process, as the larger‐sized BA spacers process a much weaker interaction with the metal halide octahedra frame. The loss of BA can facilitate the connection of metal halide octahedra in the out‐of‐plane direction of the quasi‐2D crystal and consequently lead to the formation of 3D MAPbI_3_ (*n* = ∞) at the ablation edges. Prior report on the 3D components in the LESs created by mechanical exfoliation has proposed the similar assumption.^[^ ^15^ ^]^

To further understand the correlation between PL evolution and ablation time, we perform the fs laser ablation treatment at a constant laser fluence with the different irradiation time. The in situ irradiation time dependent two‐photon excited photoluminescence (TPPL) spectra upon fs laser excitation with a wavelength of 800 nm and an ablation threshold of 11.7 mJ cm^−2^ is shown in Figure 2d. At a time of tens of microseconds after excitation, the low‐energy peak A firstly appears, followed by the appearance of the high‐energy peak B. Figure S6 (Supporting Information) shows the ex situ irradiation time‐dependent low‐energy emission (peak A) intensity upon laser fluence of 23.4 mJ cm^−2^. The low‐energy emission is only observed at the edge of the irradiation spot and gradually increases along with the increase of irradiation time. When further extending the irradiation time to 350 ms, a PL intensity saturation is observed, indicating the laser‐induced edge is confined by the total laser focal spot size. Further exceeding the laser fluence leads to a larger spot. And increasing the laser ablation fluence will lead to a higher intensity of the edge emission (Figure S4, Supporting Information).

**Figure 3:**
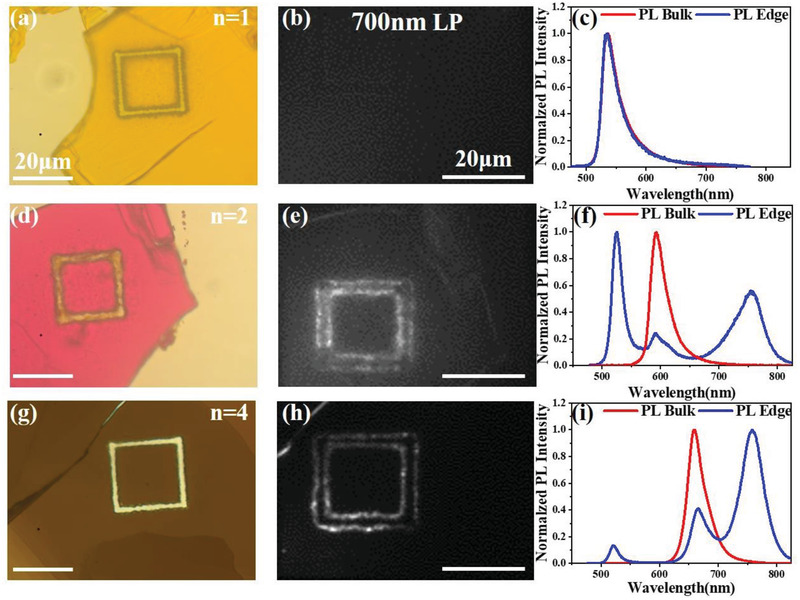
Optical images, PL images at emission channels of > 700 nm, and the representative PL spectra of the fs laser ablation induced the exfoliated 2D perovskite crystals of a−c) *n* = 1, d−f) *n* = 2, and g−i) *n* = 4, respectively. The laser ablation fluence is 48.1 mJ cm^−2^.

Similar results are also observed in *n* = 2 and *n* = 4 samples upon fs laser ablation, as shown in **Figure** **3**. Although the ratios between the three emission peaks are different, the presence of the low‐energy peak A and high‐energy peak B and their profiles are similar to those in the *n* = 3 samples. This reveals that the as‐prepared 2D perovskites (*n* > 1) decompose quickly and generate two species identically under the fs laser irradiation, which eventually lead to the observation of two emission peaks of A and B. Surprisingly, the high‐energy emission peak B is similar to the intrinsic bulk emission of *n* = 1 perovskite. Since the *n* = 1 perovskite (BA_2_PbI_4_) does not have MA component, there is no low‐energy emission peak A observed in this sample.

**Figure 4:**
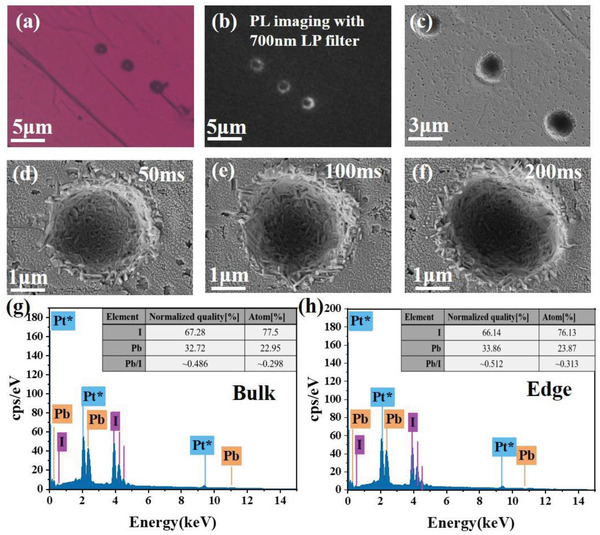
a) Optical images of (BA)_2_(MA)_2_Pb_3_I_10_ (*n* = 3) single crystal with three dots induced by fs pulse laser ablation. The three dots were created by the identical laser fluence upon irradiation time of 50, 100, and 200 ms, respectively. b) The corresponding PL (>700 nm) image upon 395 nm wide‐field excitation. c) The corresponding SEM image. d–f) The respective high‐resolution SEM images of these three dots. g,h) EDS analysis of the bulk and the laser‐induced edge regions from the *n* = 3 perovskite single crystal.

In order to verify the composition of the laser‐induced new emissive species, we conducted the SEM images along with an EDS analysis on the *n* = 3 quasi‐2D perovskite crystals upon fs laser ablation. Three laser‐induced spots are created by different irradiation time of 50, 100, and 200 ms, respectively, upon the fs laser fluence of 48.1 mJ cm^−2^. The corresponding PL intensity images collected at emission channels of >700 nm is shown in **Figure** **4**b, displaying that all the three laser‐induced spots exhibit low‐energy emission. SEM images in Figure 4c–f show that the original 2D structure has been destroyed by laser ablation, and a few 3D nanocrystal structures have formed at the edge of the spots. The EDS analysis in Figure 4g,h shows the different ratio of Pb and I elements between the bulk and edge regions. The ratio of Pb/I in the bulk position of the sample is about 0.297 which is consistent with the stoichiometric Pb/I ratio from the original chemical formula of 2D (BA)_2_(MA)_2_Pb_3_I_10_ (*n* = 3). In contrast, the ratio of Pb/I in the edge position of the sample is about 0.313 which is closer to that of 3D MAPbI_3_. Furthermore, Raman spectra (Figure S7, Supporting Information) obtained from the laser induced edge region show two typical bands (113 and 250 cm^–1^) of 3D MAPbI_3,_ which are corresponding to the vibration modes of Pb‐I octahedra cage and MA cation, respectively.^[^ ^1^, ^18^ ^]^ The variation of Pb/I ratio, the strong correlation of PL, PL lifetime and Raman spectra of 3D MAPbI_3_ suggest that MAPbI_3_ nanocrystals form at the edges of the laser ablation region and eventually give rise to the low‐energy emission of LESs.

**Figure 5:**
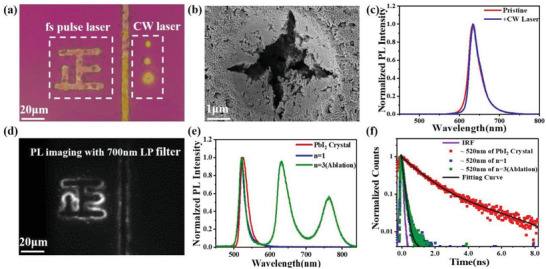
a,d) Optical and PL images of (BA)_2_(MA)_2_Pb_3_I_10_ (*n* = 3) upon 800 nm fs laser and 488 nm CW laser ablation, respectively. b) SEM images upon 488 nm CW laser ablation. c) PL spectra comparison before and after the CW Laser ablation treatment. e) PL spectra comparison of the PbI_2_ single crystal, 2D perovskite crystal (*n* = 1), and 2D perovskite crystal (*n* = 3) after fs laser ablation. *f*) Normalized PL decay dynamics at the wavelength of 520 nm from the samples in panel (e).

The origin of LESs can be further cross‐checked by the comparison study between the fs pulse laser ablation and a continuous wave (CW) laser ablation. The fs laser with a high peak power intensity can efficiently trigger an electronic heating at a high speed by two‐photon absorption with minimal thermal effect,^[^ ^19^ ^]^ while most energy of the CW laser is absorbed by the sample due to the interband transition and consequently melt the sample.^[^ ^20^ ^]^ As shown in Figure S8 (Supporting Information), the 808 nm CW laser has no laser‐ablation effect on the sample, which might be due to the lower photon energy than the bandgap of quasi‐2D perovskite crystals and thus no absorption. Then we chose a lower wavelength CW laser of 488 nm (higher energy than the bandgap of the quasi‐2D perovskites to secure the photon absorption), which is supposed be effectively absorbed by the sample and cause sufficient melting effect. **Figure** **5**a shows the optical images of quasi‐2D perovskite crystal (*n* = 3) after ablation with 800 nm fs pulse laser (121 mW as fluence of 48.1 mJ cm^−2^) and 488 nm CW laser (14.5–121 mW), respectively. The middle line and a Chinese character on the left are drawn by the fs pulse laser, while three dots on the right are written by CW laser with different powers. According to the PL image shown in Figure 5d, 488 nm CW laser irradiation does not lead to emissive LESs in the sample, which is consistent to the PL spectra in Figure 5c. The SEM image in Figure 5b also confirms the melting nature of the samples irradiated by CW laser and consequently there is no 3D MAPbI_3_ nanocrystal formed at the edges. These results suggest that melting without loss of BA molecular cannot lead to the formation of LESs.

To identify the origin of high‐energy emission peak B upon fs laser ablation, two possible species during the decomposition and reorganization process such as PbI_2_ and (BA)_2_PbI_4_ (*n* = 1) are prepared for a comparison study. As shown in Figure 5e, the emission peaks of (BA)_2_PbI_4_ (*n* = 1) and PbI_2_ single crystal locate at 518 and 525 nm, respectively. The high‐energy peak B matches well with the intrinsic emission of the *n* = 1 perovskite crystal. Furthermore, Figure 5f shows the PL lifetime dynamics of (BA)_2_PbI_4_ (*n* = 1), PbI_2_ single crystal, and quasi‐2D perovskite crystal (*n* = 3) with fs laser ablation collected at wavelength of 520 nm. The quasi‐2D perovskite crystal (*n* = 2 and 4) also share the similar PL lifetime of laser induced peak B (Figure S9, Supporting Information). The 520 nm PL lifetime is 0.18 ns which is identical to that of (BA)_2_PbI_4_ (*n* = 1) but significantly smaller than that of PbI_2_ single crystals (1.93 ns). This longer lifetime of PbI_2_ single crystal is also confirmed by the repeating measurement of different PbI_2_ single crystals in Figure S10 (Supporting Information). The strong correlation between PL spectra and lifetime of (BA)_2_PbI_4_ (*n* = 1) reveals that the high‐emission peak B induced by laser ablation is due to the formation of *n* = 1 2D perovskite crystal rather than PbI_2_.

**Figure 6:**
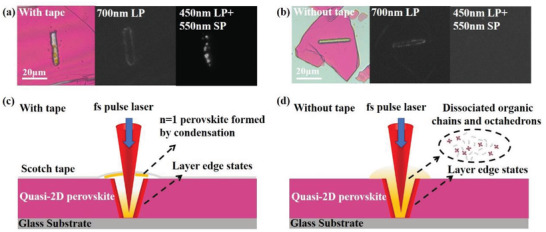
a,b) Optical images and 395 nm UV‐excited PL images collected at different emission channels of the pristine 2D perovskite crystal with fs laser ablation (*n* = 3) with and without tape isolation. c,d) The corresponding schematics of fs laser ablation mechanism.

In order to understand how the fs laser induced BA component would behave upon the fs laser irradiation (escape to air or reorganize into ordered structures), we prepare the fresh perovskite samples with and without cover tapes for comparison (**Figure** **6**a,b). PL images collected by different emission channel (low‐energy peak A and high‐energy peak B) are compared to show the variation between the samples either in sealed condition or in open air condition during fs laser ablation. As shown in Figure 6a, when fs laser irradiates the sample with a cover, the high‐energy emission (peak B) is mainly observed in the whole region of the laser irradiation. While the low‐energy emission (peak A) is only present at the edge regions (periphery of the laser ablated region). As the observed PL intensity of BA_2_PbI_4_ (*n* = 1) at edge is much lower than that at the laser irradiation region, the majority of the BA_2_PbI_4_ is most likely exist on the tapes (Figure S11a, Supporting Information). In contrast, when fs laser irradiates the sample without a cover, the high‐energy emission (peak B) even cannot be observed in the whole region of laser irradiation (Figure 6b and Figure S11b, Supporting Information). This comparison experiment reveals that the cover tape provides an enclosed chamber facilitating the re‐formation of BA_2_PbI_4_ (*n* = 1) 2D perovskites. Figure 6c,d illustrates the schematics of the fs laser ablation process in the quasi‐2D perovskite crystal at these two conditions. Upon fs laser irradiation, the organic counterparts and inorganic sublattice of quasi‐2D perovskites (*n* > 1) decompose into air due to the photochemical and thermochemical reactions induced by the high peak power of fs pulses in the focal region. Compared to MA molecular, BA spacer has a much weaker interaction with the lead halide sublattice, which is more easily released from the lattice. On the edges of the irradiation spot with lower fluence, BA spacers are partially released and the 3D MAPbI_3_ nanocrystals are formed by subsequent connection of the octahedra sublattice. In the sealed condition, the enclosed chamber blocks the evaporation of BA into air and thus the high concentration of BA in the enclosed atmosphere facilitates the assembly of the BA_2_PbI_4_ (*n* = 1) perovskites. Hence, an obviously high‐energy emission (peak B) is observed. In contrast, in the open‐air condition, BA can easily escape to the air and hardly form the BA_2_PbI_4_ (*n* = 1) perovskites. As a result, there is no observation of high‐energy emission (peak B) in this condition. While for both cases, as soon as fs laser release the BA from the lattice, the residual MA and inorganic octahedra further shrink the lattice to a 3D structure in the irradiation edge, which explains the low‐energy emission of LESs in both samples.

## 3 Conclusion

In summary, we proposed a potential approach of fs laser ablation to write LESs with highly designable capability in quasi‐2D (BA)_2_(MA)_n−1_Pb_n_I_3n+1_ (*n* > 1) single crystals. A series of comparative experiments on optical and morphological properties of the samples before and after fs pulse laser and CW laser ablation have been conducted. The results show that the LESs located at the edges of irradiation trace originate from the formation of the 3D MAPbI_3_ nanocrystals. These 3D MAPbI_3_ nanocrystals result from pulse laser induced releasing of BA spacers. Sealing of the irradiated film can facilitate the generation of LESs on the edges and induce additional high‐energy states by the formation of (BA)_2_PbI_4_ (*n* = 1) within the irradiation region. So far, most prior LESs from literatures are randomly distributed and remains challenging to pattern or program. This work provides the solution to this, showing that fs laser ablation can be a simple and efficient technique to design and write the emissive LESs with high spatial resolution and high controlling preciseness. This may open more opportunities for developing high‐performance quasi‐2D perovskite optoelectronics.

## 4 Experimental Section

### Chemicals

Lead(II) oxide (>99.0%), hydroiodic acid (57%), hypo‐phosphorous acid solution (50 wt%), and *n*‐butylamine (99.5%) were obtained from Sigma‐Aldrich. Methylammonium iodide (99.5%) was obtained from MACKLIN. All the chemicals were used as received.

### Synthesis of Quasi‐2D (BA)_2_(MA)_n‐1_Pb_n_I_3n+1_ Perovskite Single Crystals

The quasi‐2D (BA)_2_(MA)_n‐1_Pb_n_I_3n+1_ (*n* = 1, 2, 3, 4) perovskite single crystals were synthesized according to the previously reported method.^[^ ^16^ ^]^ 1.126 g lead(II) oxide (2.4 mmol) was firstly dissolved in the mixed solution of 5 mL hydroiodic acid and 850 µL hypo‐phosphorous acid. With heating up to 100 °C, the mixture was continuously stirred until a pale‐yellow solution was gained. Meanwhile, in another vial, methylammonium iodide (MAI) and *n*‐butylamine (BA, total ammonium of 5 mmol) in different molar ratios were slowly added into 3 mL hydroiodic acid with an ice bath. Since the transparent solution was obtained, the ammonium precursor was added into the lead solution with stirring at a lifted temperature. Then, the mixture was cooled down at a rate of 0.5 °C/h. Finally, the crystallized crystals were dried at 40 °C in a vacuum oven.

### Preparation of Exfoliated Quasi‐2D (BA)_2_(MA)_n‐1_Pb_n_I_3n+1_ Perovskite Single Crystal Flakes

Scotch tape (3M) was applied to exfoliate quasi‐2D perovskite single crystal. Briefly, a piece of the sample was transferred onto the adhesive side of the tape. After folding the tape with a blank position facing the single crystal, the two sides of the tape were separated to exfoliate the single crystals. After repeating this process several times, the adhesive side of tape was pressed on a piece of microscope cover glass (24 × 50, Fisherbrand) and the single crystal flakes could be observed by microscopy (Olympus IX71).

### SEM and Raman Measurement

The SEM images along with an EDS analysis were obtained from Carl Zeiss, ZEISS Gemini 500, which has a secondary electron detector and a backscattered electron detector (InLens). The sample was attached to a conducting substrate, and the platinum metal coating was sputtered onto the sample before measurement. The Raman spectra were collected by an inVia Renishaw spectrometer with a 785 nm laser for excitation.

### Time‐Resolved PL Spectroscopy and Imaging Measurement

Time‐resolved PL spectroscopy and imaging measurements were performed by a home‐build system, which mainly includes fs pulsed laser, an inverted fluorescence microscope, and a spectrometer integrated with Time‐Correlated Single Photon Counting (TCSPC) detection. The excitation laser pulses were generated from a wavelength‐tunable femtosecond oscillator (Coherent Chameleon) with 200 fs pulse width and a repetition rate of 80 MHz. The excitation laser was introduced into the microscopy (Olympus IX71) and focused on the sample via an objective lens (60×). The optical image was taken by two Scientific CMOS cameras (PCO.panda 4.2 & Sony Starvis IMX226). Short‐pass filters (for two‐photon excitation) or long‐pass filters (for one‐photon excitation) were placed in the detection path to cut off the excitation laser before measuring. The sample was placed on a 3D nano‐translation stage (Physik Instrumente, P‐525) for varying the sample position precisely. The collected emission was further imported into a spectrometer (Andor Kymera‐328i) with two output ports. One port was connected with a charge‐coupled device (CCD) camera (Andor iDus420) for the spectra measurement. The other one was integrated with a photomultiplier detector (PicoQuant PMA182) which was associated with TCSPC (HydraHarp 400) for transient PL measurement.

### Laser Ablation

An 800 nm femtosecond pulsed laser (Coherent Chameleon, 200 fs pulse width and 80 MHz repetition rate) as well as a 488 nm CW laser (CVI Melles Griot) were employed for laser ablation measurement. The ablation occurs when fs laser power was increased beyond 29.3 mW. Both of them were focused onto the sample with a radius of 1–2 µm or even smaller via an objective lens (60× or 100×). A precision electronic shutter (Hengyang, GCI‐73) was used to control the irradiation exposure time. The ablation position was controlled by the 3D nano‐translation stage (Physik Instrumente, P‐525) for micro‐pattern writing.

## Conflict of Interest

The authors declare no conflict of interest.

## Supporting information

Supporting Information

## Data Availability

The data that support the findings of this study are available in the supplementary material of this article.
