# Supplementary material for: Designable Layer Edge States in Quasi‐2D Perovskites Induced by Femtosecond Pulse Laser
Source: Adv Sci (Weinh). 2022 May 12;9(20):2201046. doi: 10.1002/advs.202201046 (PMC9284193; doi:10.1002/advs.202201046)
Supplement: Supplementary file 1 — Supporting Information [file ADVS-9-2201046-s001.pdf]

ADVANCED SCIENCE
Open Access

Supporting Information

for *Adv. Sci.*, DOI 10.1002/advs.202201046

Designable Layer Edge States in Quasi-2D Perovskites Induced by Femtosecond Pulse Laser

Yu Miao, Zeqi Xiao, Zeyu Zheng, Da Lyu, Qin Liu, Jieyu Wu, Yongbo Wu, Xiewen Wen, Lingling Shui, Xiaowen Hu, Kai Wang*, Zhilie Tang* and Xiao-Fang Jiang**

Supporting Information

Designable Layer Edge States in Quasi-2D Perovskites Induced by Femtosecond Pulse Laser

Yu Miao, Zeqi Xiao, Zeyu Zheng, Da Lyu, Qin Liu, Jieyu Wu, Yongbo Wu, Xiewen Wen, Lingling Shui, Xiaowen Hu, Kai Wang*, Zhilie Tang*, Xiao-Fang Jiang**

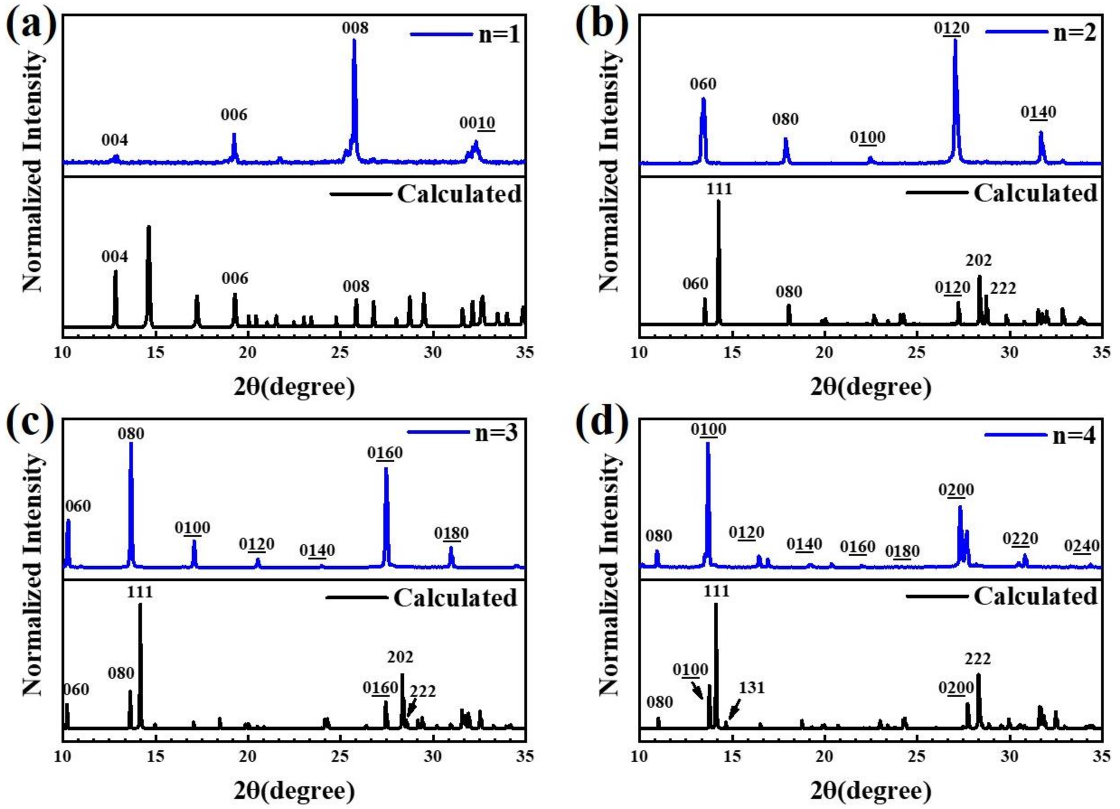

Figure S1. Comparison between XRD of quasi-2D perovskite single crystal and the simulated powder XRD data.

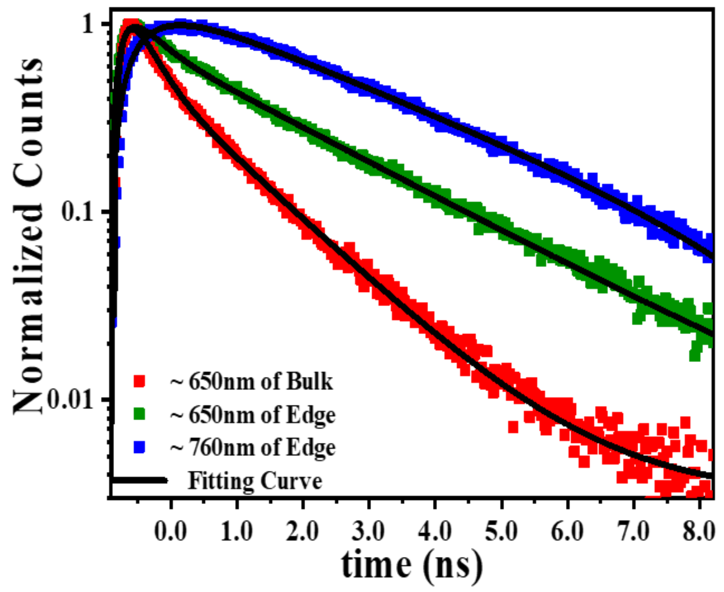

Figure S2. Normalized PL decay dynamics of the bulk (~ 650 nm) and the edge (~ 650 nm and ~760 nm) from the n=4 perovskite single crystal.

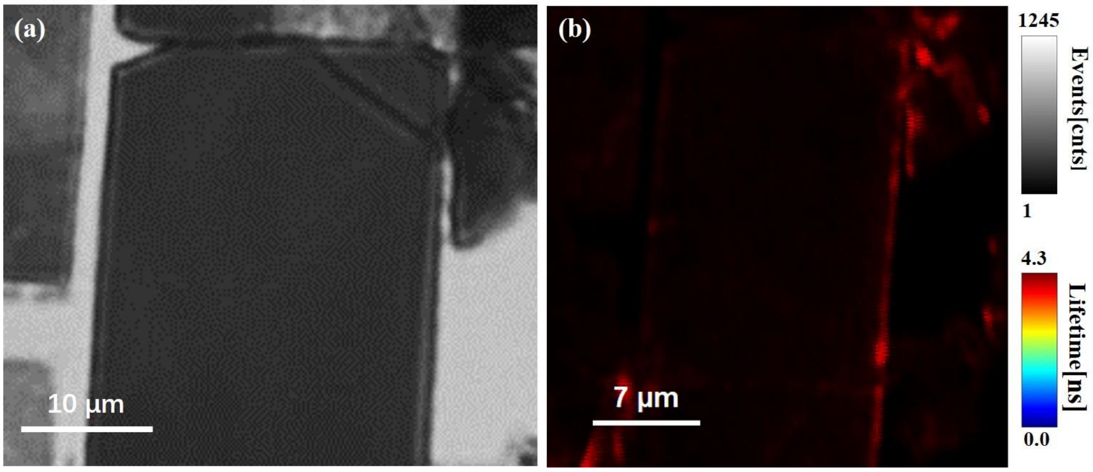

Figure S3. (a) Optical images of 2D $(BA)_2(MA)_2Pb_3I_{10}$ (n=3) single crystal. (b) Fluorescence lifetime imaging microscopy (FLIM) results of the 2D crystal in panel(a), probed at 760 nm (1.632 eV).

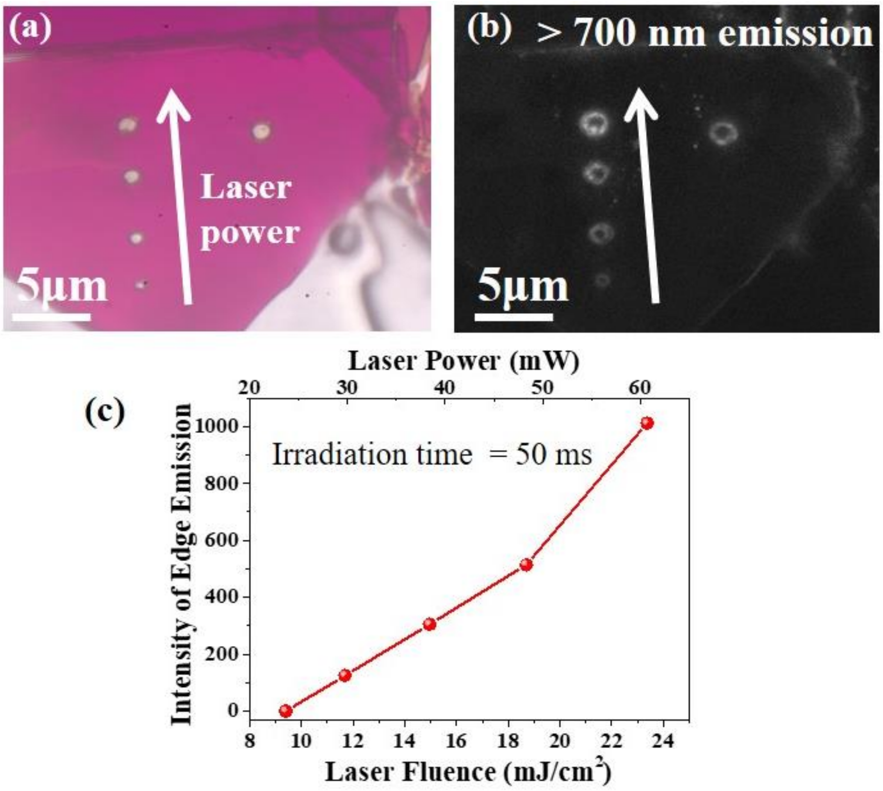

Figure S4. (a, b) Optical and PL image collected at emission channels of >700 nm of $(BA)_2(MA)Pb_2I_7$ (n=2) flakes after 800 nm fs laser ablation with different fluence upon an identical irradiation time of 50 ms. (c) The corresponding relationship between the average low-energy edge emission intensity and ablation laser fluence.

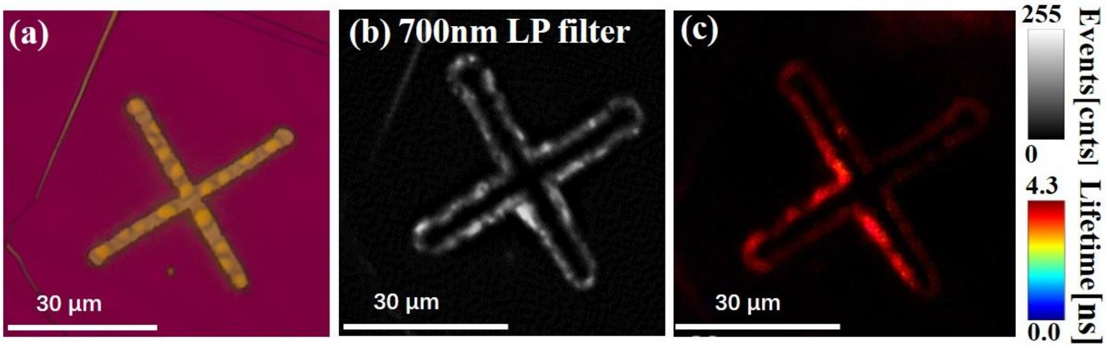

Figure S5. (a) Optical images (bright-field) of 2D $(BA)_2(MA)_2Pb_3I_{10}$ (n=3) single crystal with laser ablation pattern. (b) The corresponding PL images collected at emission channels of >700 nm upon 395 nm wide-field excitation. (c) FLIM result probed at 760 nm (1.632 eV).

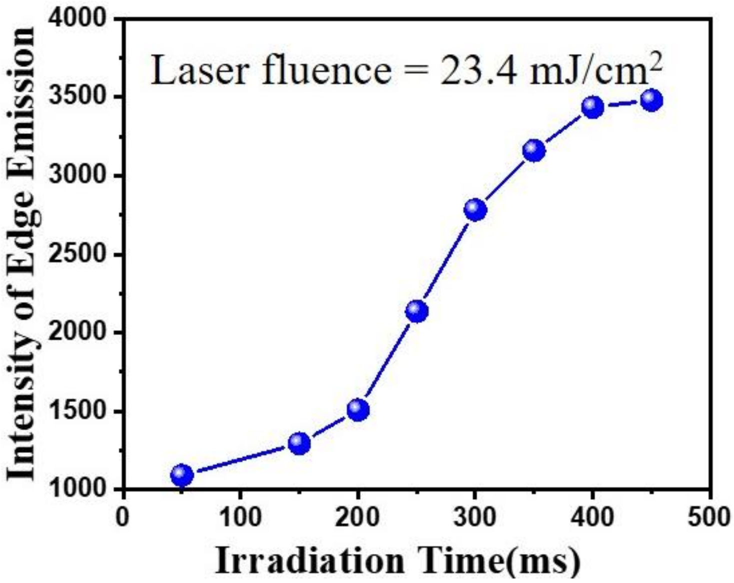

Figure S6. The relationship between the average low-energy edge emission intensity and irradiation time upon laser fluence of 23.4 mJ/cm^2.

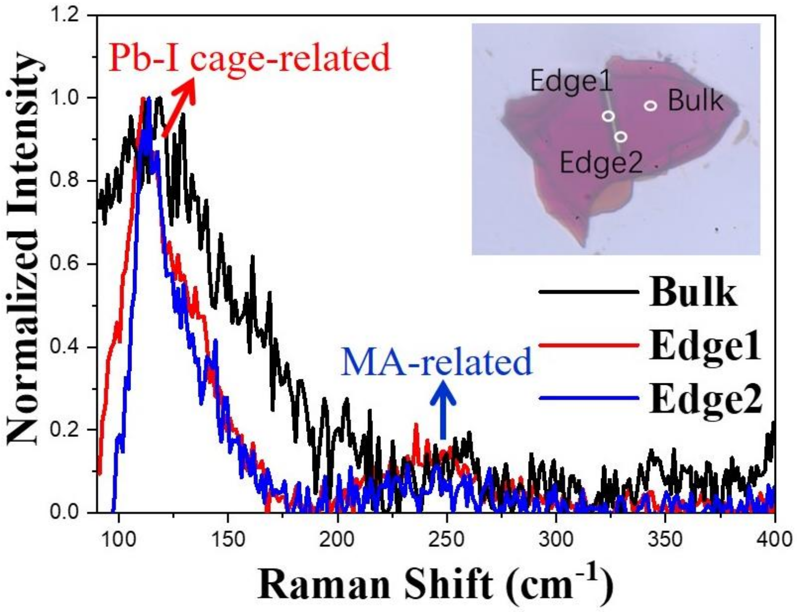

Figure S7. Raman spectra of $(BA)_2(MA)_2Pb_3I_{10}$ (n=3) flakes obtained from the bulk and fs laser induced edge region, respectively. The excitation wavelength is 785 nm.

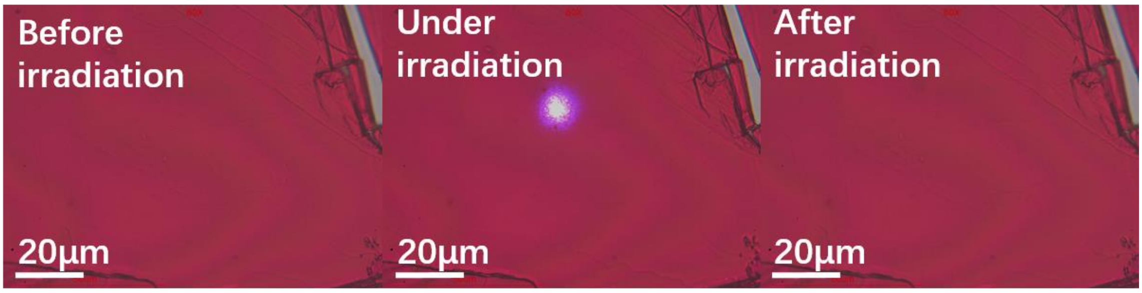

Figure S8. Optical images of the pristine 2D perovskite crystal (n=3) upon the 808 nm CW laser.

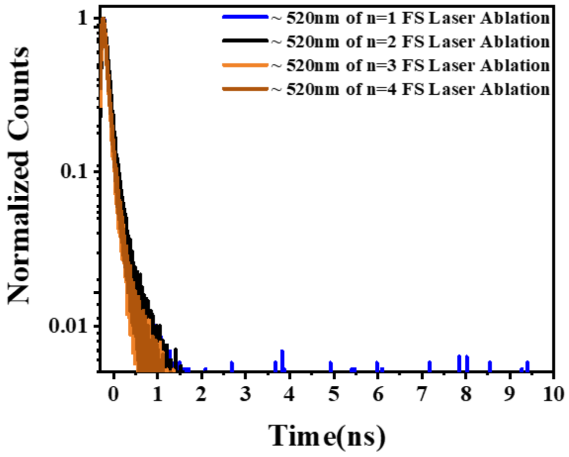

Figure S9. The PL decay dynamics of 2D perovskite crystal (n=1,2,3,4) with laser ablation collected in the emission channels of 520 nm.

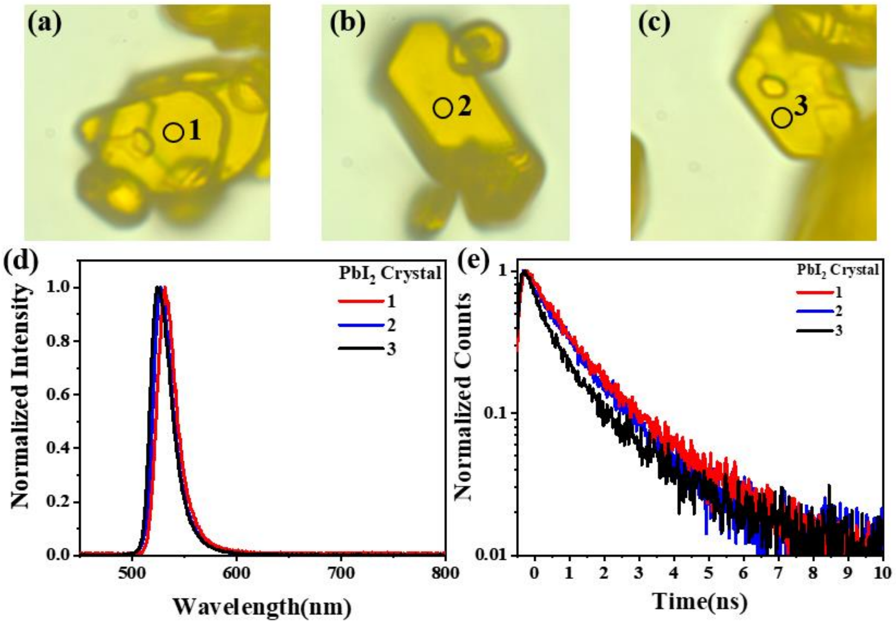

Figure S10. (a) (b) (c) Optical images of PbI_2 single crystal. (d) Comparison of PL spectra of the different PbI_2 single crystal. (e) Comparison of the normalized PL decay dynamics of the different PbI_2 single crystal.

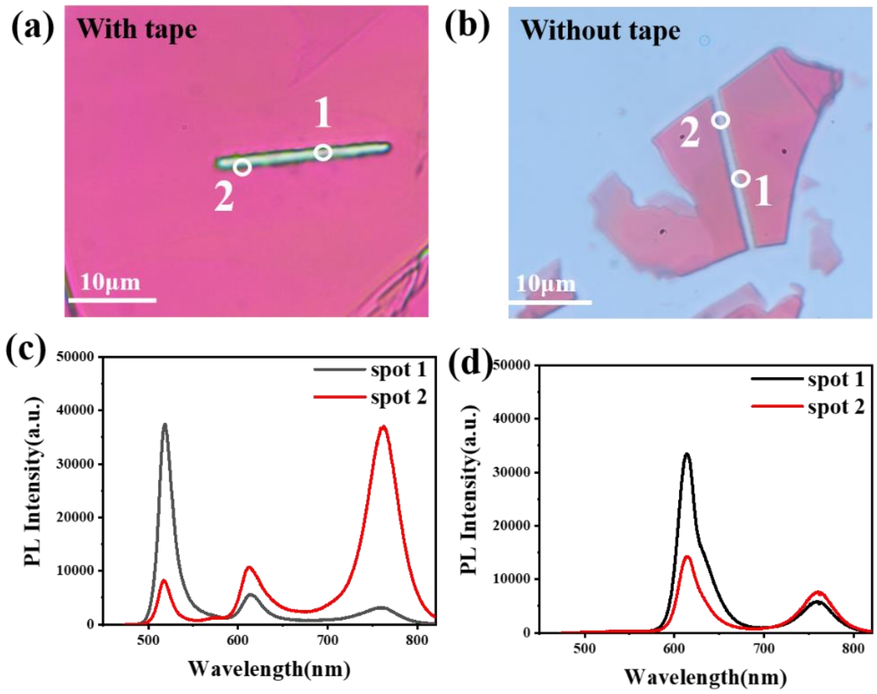

Figure S11. Optical images and position-dependent PL spectra of the pristine 2D perovskite crystal (n=3) upon fs laser ablation with and without Stoch tape isolation.
